# Processable Bio-Based Polybenzoxazine with Tunable Toughness and Dielectric Properties

**DOI:** 10.34133/research.0745

**Published:** 2025-06-24

**Authors:** Jiale Li, Meng Liu, Yuan Liu, Peng Zhao, Yuhan Lou, Zhiqian Meng, Xiaoxue Song, Zhenle Hu, Yongzhuang Liu, Haipeng Yu

**Affiliations:** ^1^ Key Laboratory of Bio-based Material Science and Technology of Ministry of Education, State Key Laboratory of Utilization of Woody Oil Resource, Northeast Forestry University, Harbin 150040, China.; ^2^ College of Home and Art Design, Northeast Forestry University, Harbin 150040, China.

## Abstract

There is remarkable demand for bio-based specialty resins such as benzoxazine thermosets, but they are brittle and difficult to process. This study reports the synthesis of a processable bio-based polybenzoxazine resin via the copolymerization of rigid and soft benzoxazine dimers synthesized from bio-based phenols. The polybenzoxazine copolymer demonstrated excellent thermoplasticity and a tunable toughness in the range of 9.0 to 24.1 MJ/m^3^. The incorporation of soft segments and dynamic ester bonds in the polybenzoxazine notably improved its thermoplasticity compared with traditional thermosetting benzoxazine resins. The polybenzoxazine copolymer also demonstrated a dielectric constant of 2.99 and a dielectric loss of 0.019 at 3 GHz, as well as a high breakdown voltage of 27.2 kV/mm. This research highlights the promising mechanical and thermal properties of the resulting bio-based resin, as well as its tunable dielectric properties, making it a competitive candidate for various high-performance applications in the polymer industry.

## Introduction

Specialty resins show exceptional performance of high thermal resistance, mechanical strength, and dimensional stability, making them instrumental in advanced applications [1,2] in the transportation and aerospace industries, as well as for the production of composites [3,4]. However, many of these resins rely on petroleum-based monomers that pose risks to human health and the environment. Due to growing emphasis on green and sustainable development, the creation of bio-based resins with equivalent performance has become important [5–8].

**Fig. 1.**
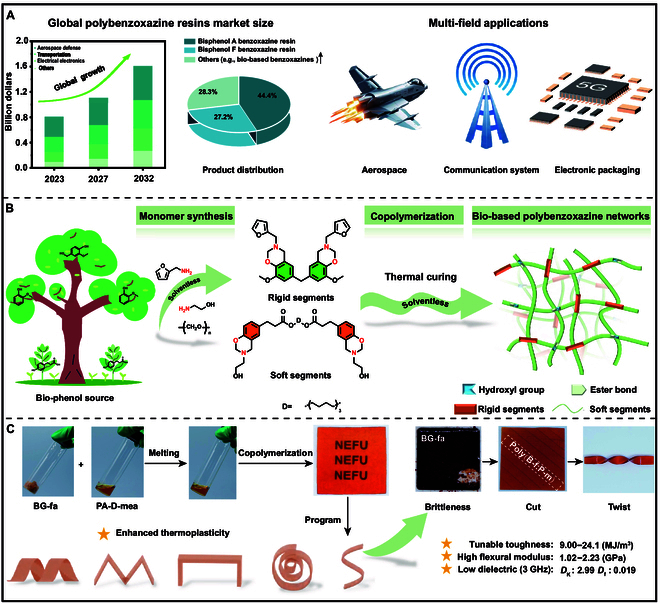
(A) Global demand and market trends for polybenzoxazine resins, along with their various applications [9]. (B) Design of processable bio-based polybenzoxazine networks achieved via the copolymerization of rigid and flexible dimeric benzoxazine segments. (C) Solvent-free copolymers of bio-based polybenzoxazines, emphasizing their tunable mechanical properties, enhanced thermoplasticity, and exceptional dielectric properties.

The demand for polybenzoxazines is projected to continue to grow for the foreseeable future (Fig. 1A) because they share many of the advantages of typical specialty resins [9], especially their molecular design flexibility. The basic principle of polybenzoxazine synthesis is rooted in the Mannich reaction; benzoxazine precursors are synthesized using adjacent available phenolic sources, formaldehyde as a methylene donor, and amines. Molecular design strategies, including chemical structures, interlinkages, and functional groups of the phenol sources, as well as amines, enable benzoxazine precursors to exhibit highly tunable and versatile properties. Moreover, the in situ ring-opening and waste-free polymerization of benzoxazine imparts low curing shrinkage and cost-effectiveness, and eliminates the need for curing agents [10]. The applications of polybenzoxazines are vast, especially in solid-state batteries, aerospace materials, and electronic packaging (Fig. 1A) [11–14]. Nevertheless, polybenzoxazines also have limitations, including high curing temperatures, increased brittleness after curing, difficult reprocessing, and mainly petroleum-based components.

To address these challenges, previous work has focused on several approaches. Researchers have introduced bio-based components and have structurally modified benzoxazine monomers to enhance their properties and expand their applications. For instance, Zhu et al. utilized a mechanically interlocked supramolecular polyrotaxane to modify the hydrogen bonding and cross-linking network of a polybenzoxazine. This approach greatly improved the ductility and fracture toughness of the polybenzoxazine without a noticeable drop in its Young’s modulus [15]. Zhao et al. introduced 2-ureido-4[1H]-pyrimidone to construct quadruple hydrogen bonding structures and a flexible alkane benzoxazine resin. Its strength, processing window, and processability were improved [16]. Incorporating softer segments by carefully selecting phenolic sources can mitigate the inherent brittleness of traditional benzoxazine networks [17]. By copolymerizing rigid and flexible benzoxazine dimers, researchers have created materials with a balance of mechanical robustness and enhanced ductility. This approach allows the mechanical properties to be fine-tuned to meet specific application requirements while expanding the processing window [18–21]. The introduction of dynamic covalent bonds, such as imines or transesterification linkages, can improve the reprocessing of benzoxazine resins by reversibly dissociating and re-forming under heat or stress. This facilitates the reshaping and recycling of these materials without notable degradation in their mechanical properties. These enhancements improve the materials’ lifetime and also contribute to sustainability by reducing waste [22–24]. Incorporating reinforcements, such as cellulose nanofibers or carbon fibers, into polybenzoxazine matrices can greatly enhance their strength and toughness [25,26]. The hybridization of bio-based fillers with synthetic resins shows great promise for advancing the functionality of benzoxazine resins. Yang et al. [27] synthesized an entirely bio-based benzoxazine resin using vanillin, erythritol, furfurylamine, and benzaldehyde, demonstrating a closed-loop process for chemical recycling. Lu et al. [28] developed a molecular design strategy that incorporated oxazine rings into bio-benzoxazine monomers to achieve thermally stable and intrinsically flame-retardant thermosets. These studies indicate that the fabrication of bio-based polymers via monomer design and the incorporation of dynamic bonds may offer solutions to the aforementioned issues [29].

Though various strategies were employed to enhance the thermal stability, mechanical performance, and functionality of polybenzoxazines, including phenol substituents regulation, functional amines selection, dynamic/vitrimeric bonds design, and multi-component copolymerization [30,31], the strategy of designing rigid and soft bio-based benzoxazine dimers and copolymerizing to achieve performance-tunable and dimension-processable polybenzoxazine is not reported to the best of our knowledge. This study aims to develop a novel bio-based polybenzoxazine resin with adjustable rigidity, toughness, flexibility, and thermoplasticity. Initially, bisphenols were synthesized from bio-derived phenol monomers, then hemicellulose-derived amines and paraformaldehyde were employed in a solvent-free process to create the rigid segments of the polybenzoxazine. Phloretic acid was employed as a phenol source to synthesize the flexible segments of the polybenzoxazine using a solvent-free method. The inherent molecular design flexibility of ethanolamine provided a lower curing temperature while enhancing the thermoplasticity of the copolymerized bio-based polybenzoxazine resin (Fig. 1B). By copolymerizing these 2 components, we addressed the notable brittleness of rigid segments and demonstrated that a copolymer ratio of 1:3 provided the lowest dielectric properties, showing substantial potential for applications in electronic packaging materials. The resulting toughness surpassed that of most petroleum-based polybenzoxazines. In terms of reprocessing performance, the copolymerized polybenzoxazine could be molded into various shapes while maintaining its stability, thereby meeting the requirements of various high-grade, precision, and advanced applications (Fig. 1C). This study synthesized a bio-based polybenzoxazine primarily using natural raw materials, providing a path for innovative designs of other environmentally friendly functional materials.

## Results and Discussion

### Solventless synthesis of bio-based benzoxazine

**Fig. 2.**
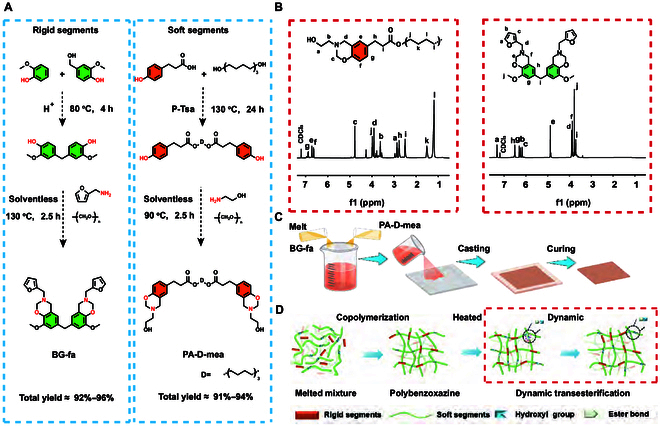
Synthesis, characterization, and polymerization of the benzoxazine resin. Synthesis of soft (PA-D-mea) and rigid (BG-fa) benzoxazine dimers (A) and their structural characterization via ^1^H NMR (B); the melting and thermal polymerization process of polybenzoxazine resin (C) and a schematic diagram of the resulting network (D).

Soft and rigid bio-based benzoxazine dimers were synthesized using a solventless process, resulting in a total yield exceeding 90%, as shown in Fig. 2A. This method enhanced the efficiency of the synthesis and also aligned with the principles of green chemistry by eliminating solvent use [32,33]. To confirm the structure of the synthesized dimers, ^1^H nuclear magnetic resonance (NMR) and 2-dimensional (2D) NMR were employed. In Fig. 2B, the ^1^H NMR spectra show characteristic peaks corresponding to the protons in the benzoxazine structure of BG-fa (the benzoxazine dimer of guaiacol-based bisphenol) and PA-D-mea (the benzoxazine dimer of phloretic acid-based bisphenol), indicating a successful synthesis. These peaks were integrated to quantify various protons, which confirmed the formation of the desired dimers. The 2D NMR spectra provided additional insights into coupling and correlations between protons, and the main by-products were speculated to facilitate a detailed understanding of the molecular structure. Correlations within the 2D spectra confirmed the presence of the functional groups essential to the benzoxazine structure (Figs. S1 and S2).

From the perspective of molecular design, the selection of ethanolamine and furfurylamine as complementary amine sources is governed by the following considerations: First, the incorporation of ethanolamine markedly reduces the curing temperature during copolymerization [34], while its amine group engages in dynamic transesterification processes, imparting essential processability to the polybenzoxazine system [35]. Second, the adoption of furfurylamine arises from the practical demands of benzoxazine applications, as its distinctive molecular architecture markedly attenuates the material’s dielectric constant. Critically, to address the limited cross-linking sites in BG-fa copolymerization, the introduction of furfurylamine further promotes the formation of a robust hydrogen-bonding network during internal copolymerization [36].

The rigid and soft benzoxazine dimers of BG-fa and PA-D-mea were melt-blended, cast, and thermally cured under solvent-free conditions to synthesize a benzoxazine resin (Fig. 2C) with a typical 3-dimensional (3D) network structure, as shown in Fig. 2D. Additionally, the flexible alkyl chains and dynamic ester bonds in PA-D-mea are expected to enhance the toughness and processability of the polybenzoxazine resin.

### Analysis of the segmental structure of polybenzoxazine resin

**Fig. 3.**
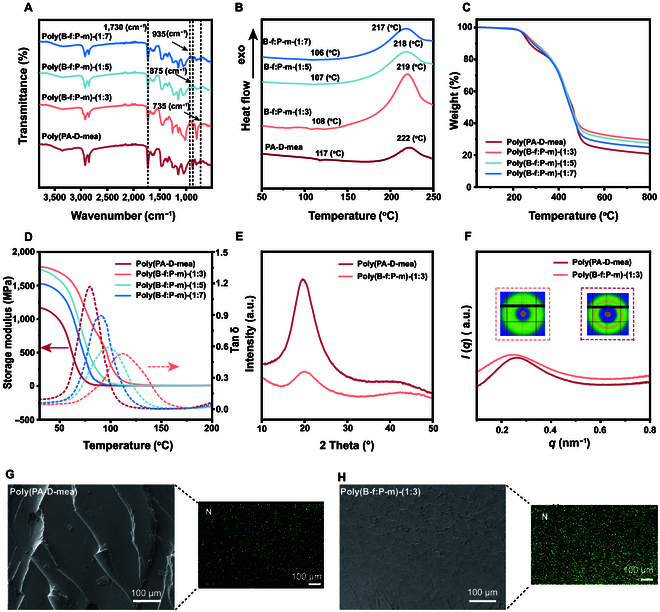
Analysis of chain segments and structural characteristics of polybenzoxazine resin. Characterization of (A) functional group characteristics, (B) melting and curing temperature, (C) thermal stability, and (D) dynamic thermomechanical performance of materials after copolymerization with different proportions; the crystallization characteristics of poly(PA-D-mea) and poly(B-f:P-m)-(1:3) (E, XRD analysis; F, SAXS analysis) and characterization of SEM and EDS mapping (G and H).

The BG-fa and PA-D-mea were copolymerized at different ratios, and then Fourier transform infrared (FTIR) spectroscopy was performed to confirm the chemical structure of the polybenzoxazine copolymers. As shown in Fig. 3A, a typical characteristic peak of benzoxazine appeared at 935 cm^−1^ [37], and the disappearance of this peak indicated that the material completed ring opening. Compared with the unpolymerized material, a new peak appeared at 875 cm^−1^, demonstrating the formation of a *para*-substituted aromatic ring in the monomers and confirming the formation of a cross-linked network. The FTIR spectra of PA-D-mea and BG-fa are included in Fig. S3. After ring-opening, polybenzoxazine substituents were typically *ortho* and *para* to the hydroxyl groups. However, in BG-fa, the *ortho* and *para* positions were occupied by methoxy and methylene groups, which is the main reason for the lower cross-link density and greater brittleness of BG-fa. Nonetheless, in the copolymer subjected to high temperatures, a obvious reduction in the peak at 735 cm^−1^ was observed, suggesting that the cross-linking of penta-substituted aromatic rings occurred, resulting in a denser cross-linked network. The polymerization of the main product of polybenzoxazine was inferred by FTIR spectroscopy (Fig. S4).

Differential scanning calorimeter (DSC) measurements were used to provide insights into the thermal transitions of the polybenzoxazine copolymers (Fig. 3B), which indicated that the maximum temperatures for PA-D-mea copolymers at different ratios (1:3, 1:5, and 1:7) ranged between 217 °C and 219 °C (Table S1 and Fig. S5), which primarily reflects the ring-opening polymerization of benzoxazine. The peak curing temperature of the rigid benzoxazine segment (BG-fa) was determined to be 238 °C. In contrast, the copolymer incorporating flexible benzoxazine segments exhibited a peak curing temperature approximately 20 °C lower than that of the pure rigid benzoxazine BG-fa. This reduction originated from the introduction of adjacent aliphatic hydroxyl –OH groups in the flexible benzoxazine dimer. The solvent-free method produced oxazolidine and phenolic or aliphatic −OH groups in the benzoxazine structure, which activated polymerization at lower temperatures [35]. Furthermore, compared with other bio-based benzoxazines or petroleum-based benzoxazines (220 to 260 °C), the polymerization temperature of the copolymers was initiated notably earlier [38]. The DSC analysis reveals that this benzoxazine copolymer exhibits a distinctive dual-stage processing behavior. The integration of flexible and rigid segments reduces the melting temperature of the copolymer precursor during the initial endothermic melting stage 100 to 120 °C, thereby enhancing system fluidity and enabling efficient processing and molding. In the subsequent oxazine ring-opening curing stage 150 to 220 °C, the copolymers initiate cross-linking until achieving full curing. Based on the DSC findings, the optimal curing and processing parameters were established for subsequent investigations. The copolymer demonstrates distinctly separated melting and curing temperatures, establishing a defined processing window 47 to 53 °C for gas release during polymerization, as detailed in Table S1. This temporal processing temperature window possibly mitigates void entrapment during curing, aligning with observations reported in prior studies. [20]

Thermogravimetric analysis (TGA) indicated that the polybenzoxazine underwent thermal degradation temperature *T*_d5%_ exceeding 250 °C (Fig. 3C and Table S2), suggesting that the material possessed a high thermal decomposition temperature. The TGA curve demonstrated that the thermal degradation of poly(B-f:P-m) predominantly occurred in 2 stages. The first stage occurred between 230 and 370 °C, during which the mass decreased by 4% to 20%. The second stage occurred between 370 and 480 °C, where the mass decreased by 20% to 78%. The first stage was predominantly driven by the cleavage of ester bonds within the copolymer, while the second stage was primarily attributed to the rupture of Mannich bridges. The results revealed a degradation sequence initiating with ester bond cleavage, followed by Mannich bridge breakdown, findings consistent with published literature [39,40]. As the number of rigid chain segments of BG-fa increased within the copolymer, both the thermal stability and char yield improved due to the participation of furan rings in cross-linking reactions.

The glass transition temperature (*T*_g_) and loss factor of the materials were analyzed using dynamic mechanical analysis (DMA) as shown in Fig. 3D. The stiffness of the material was directly reflected by its storage modulus, while the damping characteristics of the material were represented by the loss factor. The *T*_g_ changed remarkably with different ratios of soft chain and rigid chain dimers, highlighting the tunability of the synthesized resin. As the proportion of rigid segment BG-fa increased, a copolymer ratio of 1:3 yielded the maximum storage modulus, and the *T*_g_ reached 112 °C. This was attributed to the higher incorporation of BG-fa into PA-D-mea, which resulted in a denser cross-link density of 1,321.94 mol/m^3^ (Table S3). Although the *T_g_* value in our work is lower than many literature values, it remains comparable to or higher than those of other processable polybenzoxazines. This underscores the need to balance Tg and processability during polybenzoxazine synthesis (Table S4). The amount of BG-fa was not further increased because this may increase the brittleness of the material. The enhanced *T_g_* of the copolymer is predominantly attributed to the suppression of flexible chain mobility by rigid benzoxazine segments. Through molecular design strategies, we successfully incorporated benzoxazine structural units with intrinsic rigidity into the polymer backbone. The synergistic effects of intermolecular interactions are likely contributors to the notable reduction in flexible chain segment mobility. This restricted molecular motion ultimately translates to a remarkable increase in the macroscopic *T_g_* of the system. Furthermore, the gel content test for both poly(PA-D-mea) and each copolymer (1:3, 1:5, and 1:7) further confirmed the predominant formation of a cross-linked polymer network, with less than 3% soluble fraction within the polymer matrix for each sample (Fig. S6 and Table S5).

The x-ray diffraction (XRD) patterns in Fig. 3E reveal distinct crystallographic reflections at 2*θ* = 20°. PA-D-mea exhibited greater crystallinity due to the ordered structure of the segments, which was influenced by strong hydrogen bonding between chains and the internal hydrogen bonding of the benzoxazine. However, after the introduction of BG-fa, the incorporation of phenyl and furan rings increased the distance between the flexible segments. This disrupted the original ordered structure of the PA-D-mea segments and weakened the crystallinity of the flexible segments, which was expected. Small-angle x-ray scattering (SAXS) analysis in Fig. 3F provides further insights into the structural characteristics of PA-D-mea and the poly(B-f:P-m)-(1:3). The SAXS data of PA-D-mea indicated a microphase periodicity of 23.78 nm, suggesting the presence of phase segregation. In contrast, poly(B-f:P-m)-(1:3) exhibited a higher periodicity of 25.52 nm (Table S6), as confirmed by a broadened shoulder peak. The incorporation of phenolic structures enhanced the rigidity of the segments, which may interfere with hydrogen bonds, thus expanding the distance of the periodic microphase-separated structures.

Figure 3G and H illustrate the microstructure and elemental distribution of poly(PA-D-mea) and poly(B-f:P-m)-(1:3), respectively. The cross-sectional morphology of the cured polymeric resin was characterized using the liquid nitrogen embrittlement method. As illustrated in Fig. 3G and Fig. S7, the scanning electron microscopy (SEM) image of the fracture surface of bulk-cured poly(PA-D-mea) resin reveals distinct fracture striations distributed across the cross-section, indicative of plastic deformation zones and confirming the material’s inherent toughness. Conversely, with the progressive incorporation of rigid BG-fa segments, the fracture striations gradually diminish, accompanied by a smoother morphological texture. This structural evolution suggests an enhanced material brittleness induced by rigid-segment incorporation. Energy dispersive spectroscopy (EDS) mapping analysis demonstrates a spatially homogeneous nitrogen distribution across the polybenzoxazine samples, confirming that both dimer species sustain uniform dispersion during copolymerization without notable single-molecule aggregation.

### Mechanical and processing properties of bio-based polybenzoxazine

**Fig. 4.**
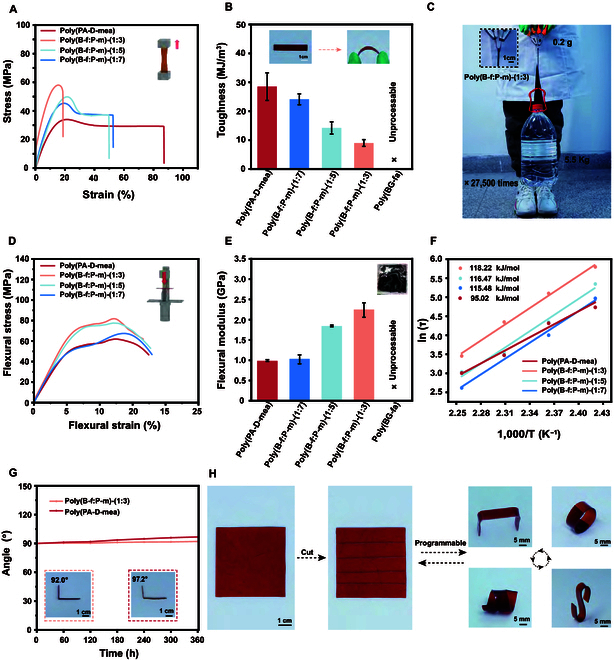
Mechanical properties and processing characteristics of bio-based benzoxazines. (A) Mechanical tensile performance of benzoxazine at different copolymerization ratios and (B) toughness values; (C) load-bearing capacity of poly(B-f:P-m)-(1:3); (D) bending strength and (E) bending modulus; (F) activation energy of the material and (G) mechanical forming stability; (H) processing and shaping characteristics of the materials.

The mechanical evaluation of the copolymerized bio-based benzoxazine revealed a notable increase in tensile strength to a maximum of 56.1 MPa (Fig. 4A). This enhancement was attributed to the incorporation of a greater number of rigid dimers during copolymerization. As the rigidity of the copolymer increased, the tensile strain decreased. This behavior aligns with the expected outcomes that introducing stiffer molecular segments into the polymer matrix enhanced its deformation resistance. Despite the increased tensile strength, the toughness decreased as the proportion of rigid dimers increased. However, the polybenzoxazine resin maintained a tunable and high toughness in the range of 9.0 to 24.1 MJ/m^3^ (Fig. 4B). A quantitative comparison of the mechanical performance in this work with reported data in the literature demonstrated that the strength and elongation of poly(B-f:P-m)-(1:3) were comparable to, or even surpassed, those of most existing benzoxazine resins (Table S4) [40–42]. Notably, the synthesized poly(B-f:P-m)-(1:3) copolymer could lift a weight 27,500 times its own mass, demonstrating its exceptional mechanical properties, as illustrated in Fig. 4C. This balance between properties—a higher tensile strength and toughness retention—indicates the potential of this bio-based resin for various applications where both properties are critical.

Figure 4D and E illustrate the flexural strength and modulus of the benzoxazine copolymers (Table S7). The results indicate that the incorporation of BG-fa during copolymerization significantly enhanced the bending strength, with the strength increasing as the proportion of BG-fa increased. The poly(B-f:P-m)-(1:3) copolymer exhibited the highest bending strength of 82.6 MPa and a bending modulus of 2.2 GPa. In contrast, materials prepared only from the rigid dimer poly(BG-fa) were difficult to shape. This study proposes an innovative strategy to tune the toughness and strength of these bio-based benzoxazine resins via the copolymerization of both rigid and flexible monomers. The stress relaxation characteristics of the copolymerized polybenzoxazine resin were analyzed to provide insights into their temperature-dependent mechanical properties (Fig. S8 and Table S8). The data revealed that the presence of rigid segments helped regulate the mechanical properties, which impacted the material’s response under sustained loads. Figure 4F shows that the activation energy of the poly(B-f:P-m)-(1:3) copolymer reached 118.22 kJ/mol, indicating more restricted molecular motion due to the presence of rigid segments.

Figure 4G demonstrates the mechanical stability of the poly(B-f:P-m)-(1:3) copolymer. After thermal processing to maintain a specific angle of 90°, the copolymer exhibited only a 2° change when left at room temperature for 360 h. In contrast, the angle change of the poly(PA-D-mea) copolymer without rigid segments reached 7.2°, underscoring the important role of the rigid segments in the material. Compared with traditional benzoxazine thermosets, the materials developed in this study exhibited better cutting and thermoplastic processing characteristics, as shown in Fig. 4H. The material could be cut into strips and thermoplastically molded into various 3-dimensional curved structures. This versatility was attributed to the controlled flexible segments and the dynamic network formed by ester bonds during preparation. A key advantage of the dynamic ester linkages in polybenzoxazine lies in its intrinsic tertiary amine substructures enabling in situ catalysis of the transesterification process [43,44], as shown in Fig. S9, thereby imparting processability to the bio-based benzoxazine resin [45]. The recyclability of the bio-based polybenzoxazine was investigated, as illustrated in Fig. S10. The synthesized polymer, specifically poly(B-f:P-m)-(1:3), could be efficiently recycled via hot pressing at 160 °C for 2 h following either physical grinding or acetic acid degradation. The mechanical performance of polybenzoxazine after successive recycling cycles was assessed, as depicted in Figs. S11 and S12 and Table S9. Notably, the tensile strength and elongation of the first reprocessed sample subjected to mechanical crushing and hot pressing retained 72.5% and 86.1% of the original values, respectively, compared to the freshly prepared sample. In contrast, the second reprocessed sample displayed a marked reduction in retention, with strength and elongation declining to 42.6% and 57.2%, respectively. This decline likely stems from partial disruption of the covalent cross-linking sites during mechanical crushing, despite the persistence of dynamic ester linkages. Remarkably, after supplementing 20 wt% (B-f:P-m)-(1:3) precursors into the second mechanically crushed material, the retention of strength and elongation rebounded to 80.4% and 81.3%, respectively, suggesting that precursor replenishment may facilitate reconstruction of the cross-linking network. These findings highlight the promising recyclability of the bio-based polybenzoxazine developed in this work.

To evaluate the performance of the prepared polybenzoxazine in composite structures, the (B-f:P-m)-(1:3) resin was immersed into carbon fiber, bamboo fiber, and glass fiber matrices and cured via hot pressing at 160 °C for 2 h. The results macroscopically exhibited good compatibility of the resin with the fiber matrices (Fig. S13), albeit the properties of the composites remain to be determined in future studies. To determine if the resin could be recycled from the fiber matrix of the composite, a carbon fiber/poly(B-f:P-m)-(1:3) composite was selected, as shown in Fig. S14. It can be observed that after acid degradation at room temperature for 2 h, the carbon fiber matrix could be easily separated after washing, while the resin could also be recovered through evaporation and filtration. This preliminary investigation of composite synthesis and recycling demonstrates the potential application of polybenzoxazine in closed-loop composite materials; however, detailed characterization and performance testing of the composites are still necessary in future studies.

Meanwhile, the bio-based polybenzoxazine contains ester bonds, which are from the soft segmental dimeric benzoxazine (PA-D-mea). The self-healing characters of poly(PA-D-mea) and poly(B-f:P-m)-(1:3) were performed, and it was found that the self-healing efficiency of poly(PA-D-mea) was 71.7% after heating at 150 °C for 4 h, while the value for poly(B-f:P-m)-(1:3) was 45.5% (Fig. S15). These observations revealed that the rigid benzoxazine dimers in the copolymer influence the self-healing properties, possibly due to enhanced cross-linking density or restricted chain mobility, which restrains the self-healing efficiency [46]. In comparison with previous studies that used benzoxazine precursor design or dynamic/vitrimeric bond design to improve the processability [24,47,48], this study employed the molecular design of bio-based dimeric benzoxazine and copolymerization of rigid and soft dimers for preparing tunable and processable bio-based polybenzoxazine. The obtained polybenzoxazine demonstrated obviously improved thermal plasticity and could be processed into various 3D shapes; meanwhile, tunable mechanical property was achieved, which showed more advantages compared to other strategies.

The mechanical properties and processability of the bio-based benzoxazine resins were tunable, representing a significant advancement in the design of specialty resins. The tensile strength, toughness, and the stress relaxation behavior make this material a potential contender for applications requiring mechanical properties and sustainability.

### Dielectric properties of bio-based polybenzoxazine

**Fig. 5.**
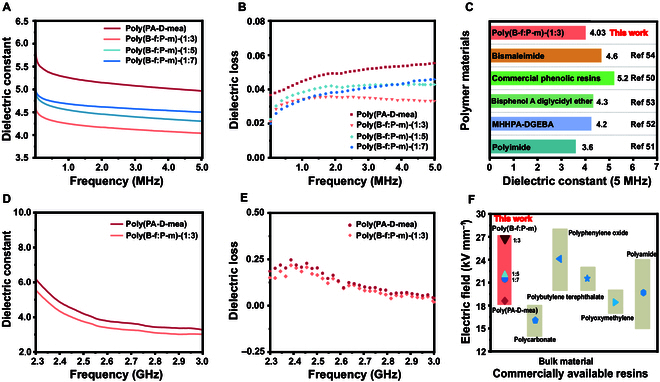
Dielectric properties of different proportions of copolymerized benzoxazine resins. (A) Dielectric constant and (B) dielectric loss of the material at frequencies in the range of 0 to 5 MHz. (C) Comparison of the dielectric constant of poly(B-f:P-m)-(1:3) at 5 MHz with other commonly used dielectric resins. Comparison of (D) dielectric constant and (E) dielectric loss of poly(PA-D-mea) and poly(B-f:P-m)-(1:3) at 2.3 to 3.0 GHz. (F) Electric breakdown performance of resins with different copolymerization ratios and a comparison with other materials.

The dielectric properties of the copolymerized bio-based polybenzoxazine were assessed across different copolymerization ratios. Figure 5A and B show that as the amount of rigid segments increased, the dielectric constant of the resin significantly decreased in the frequency range of 0 to 5 MHz. Studies demonstrate that the furan ring’s rigid framework effectively suppresses dielectric loss via dipole orientation restriction and steric hindrance mechanisms, while its π-conjugated system bolsters high-frequency response performance by establishing electron delocalization pathways. This “flexibility–rigidity balance” molecular design strategy enables precise modulation of the material’s dielectric properties [21]. This suggests that introducing rigid segments enhanced the overall dielectric properties of the material, potentially making it suitable for applications requiring lower mitigating dielectric breakdown under electrical stress [49]. The bio-based benzoxazine resin developed in this study exhibited a dielectric loss of only 4.03 at a frequency of 5 MHz, showing remarkable advantages over traditional thermosetting resins [50], such as bismaleimide phenolic resins and epoxy resins. This performance is even comparable to polyimide resins [51–54], as illustrated in Fig. 5C. At a high frequency of 3 GHz, the material also demonstrated a low dielectric constant (2.99) and low dielectric loss (0.019), as shown in Fig. 5D and E, highlighting its potential applications in communication electronic devices.

The electrical breakdown performance of the copolymerized resin was evaluated under different copolymerization ratios. The results indicated an impressive maximum breakdown voltage of 27.2 kV/mm, demonstrating its excellent electrical stability (Fig. 5F). The electric breakdown voltage could be adjusted from 18.1 to 27.2 kV/mm to meet different application requirements (Table S10). This was comparable to, or even superior to, that of traditional petroleum-based polymers, such as polycarbonate, polybutylene terephthalate, polyphenylene oxide, polyamide, and polyoxymethylene (Fig. 5F) [55]. This further confirms the potential of bio-based resins in high-voltage applications. The water resistance of bio-based polybenzoxazine resins was also analyzed (Fig. S16). The results of contact angle measurements indicated that the rigid chain segments had little effect on the hydrophobicity of the resin. The stable contact angle suggested improved water repellency, which is vital for outdoor or moisture-laden environments. The water absorption expansion rate test after 1 month confirmed the enhanced water resistance. The lower expansion upon water exposure highlighted that the rigid dimers restricted water ingress, ultimately improving the long-term dimensional stability and performance in various applications.

The dielectric properties of the bio-based polybenzoxazine resin demonstrate a promising advancement in the development of specialty materials. The low dielectric constant and loss, high breakdown voltage, and good water resistance indicated that this bio-based resin is well-positioned for various high-performance applications where electrical insulation and moisture resistance are crucial.

## Conclusion

In summary, this research prepared a novel bio-based polybenzoxazine resin with both thermoplasticity and desirable mechanical properties. The strategic copolymerization of 2 distinct benzoxazine dimers, along with the incorporation of dynamic ester bonds, allowed for the effective modulation of rigidity and flexibility within the resin matrix. The performance of the resulting material surpasses that of traditional thermosetting benzoxazine resins in terms of thermal and mechanical properties, and it also exhibited tunable dielectric characteristics. This provides a broader scope of applications in various industries. The introduction of bio-based components represents a remarkable advancement in sustainable material solutions, addressing growing environmental concerns while maintaining high-performance standards. Future work may focus on optimizing resin formulations and exploring additional applications, further establishing bio-based polybenzoxazine resins as sustainable alternatives for high-tech materials.

## Methods

### Materials

All commercially available chemicals used were analytical-grade reagents and were used as-received without further purification. Dichloromethane, petroleum ether, ethyl acetate, and concentrated sulfuric acid (98%) were purchased from Tianjin Fuyu Fine Chemicals Co., Ltd. Vanillin, guaiacol, resin acids, ethanolamine, 1,12-dodecanediol, and silica gel for column chromatography were sourced from Shanghai Yien Chemical Technology Co., Ltd. Anhydrous magnesium sulfate and paraformaldehyde were obtained from Aladdin Chemical Reagents (Shanghai, China), and deionized water was prepared in the laboratory.

### Synthesis of guaiacol-based bisphenol and its benzoxazine dimer derivative

In a 3-necked round-bottom flask equipped with a stirrer, a thermometer, and a nitrogen inlet, an excess of guaiacol (0.4 mol) and vanillyl alcohol (0.15 mol) was added. The mixture was heated to reflux at 80 °C and held for 30 min until the solution became clear. Concentrated sulfuric acid (1 wt%) was added dropwise while continuously mixing. The mixture was held at 80 °C for 4 h, then cooled to room temperature and dissolved in dichloromethane (100 ml). An appropriate amount of silica gel for column chromatography was added for purification, and then flash chromatography (petroleum ether:ethyl acetate, 80:20) was used to separate bisphenol (BG) from other isomers [6]. A white solid powder was obtained with a yield of approximately 55%. The ^1^H NMR spectrum of BG is included in Fig. S17. The obtained BG was reacted with paraformaldehyde and furfuryl amine in a molar ratio of 1:4:2 in a flask equipped with a stirrer using a solventless method at 130 °C for 2.5 h. The reaction mixture was then cooled to room temperature, yielding a light yellow solid with a total yield of 92% to 96%.

### Synthesis of phloretic acid-based bisphenol and its benzoxazine dimer derivative

Phloretic acid and 1,12-dodecanediol were placed in a 250-ml single-necked flask in a molar ratio of 2:1.2, with excess diol to ensure a complete reaction. *p*-Toluenesulfonic acid (1 wt%) was added as the catalyst, and the temperature was raised to 130 °C for 24 h and stirred at 300 rpm. After cooling to room temperature, the product was dissolved in dichloromethane, and the solution was washed with deionized water 3 to 5 times to remove the catalyst and unreacted diol. The organic solvent was dried over anhydrous magnesium sulfate and then removed using a rotary evaporator, yielding a deep yellow liquid with a yield of 90% to 95% (Fig. S18). The resulting PA-D was reacted with paraformaldehyde and ethanolamine in a molar ratio of 1:4:2 in a flask equipped with a stirrer. The mixture was stirred at 90 °C for 2.5 h at 200 rpm and then cooled to room temperature to obtain a yellow viscous liquid with a yield of 91% to 94%.

### Melting copolymerization of different benzoxazine dimers

Different ratios of copolymerized monomers were prepared. A representative copolymerization process involved placing BG-fa in a single-necked flask, heating it to 120 °C, and then adding the mixture to a sample vial containing PA-D-mea at 60 °C, where it was quickly stirred to homogenize. The viscous liquid was poured into a polytetrafluoroethylene (PTFE) mold for pre-polymerization at 120 °C for 30 min, followed by a gradient temperature increase from 130 to 180 °C at a rate of approximately 6 °C/h. This procedure yielded various proportions of the polymer. The copolymer was named poly(B-f:P-m)-(x:y), where *x*:*y* represents the molar ratio of the 2 dimers.

### Cutting and molding conditions of polybenzoxazine

Typically, the polybenzoxazine sample was cured in a PTFE mold with dimensions of 50 × 50 × 0.8 mm^3^. The cutting and shaping process of the sample was performed at 100 °C. Once the sample reached the predetermined process temperature, it was cut to the required dimensions according to the design specifications and precisely fitted, folded, and bent to match the mold shape. Finally, the processed sample was cooled naturally to room temperature to complete the molding process.

### Characterization and tests

#### Structure and chain segment characterization

^1^H NMR and 2D NMR spectra were recorded using a Bruker Avance III HD 500-MHz spectrometer with deuterated chloroform as the solvent. FTIR spectra were obtained using a Bruker VERTEX 80 V spectrometer in the wavenumber range of 400 to 4,000 cm^−1^. XRD patterns were collected using an Ultima IV x-ray diffractometer under an accelerating voltage of 40 kV and a current of 30 mA, using Cu Kα radiation. SAXS was conducted using an upgraded Xeuss system and images were captured with a Pilatus 100 K detector. The 2D SAXS patterns were integrated over a 360° azimuthal angle using Fit2D software to yield one-dimensional SAXS intensity distributions. SEM was performed using a Hitachi Regulus 8100 instrument. The thermal properties and curing behavior of benzoxazine and resin were evaluated using a TA Instruments Q20 DSC. TGA was performed with an STA 6000-SQ8 under a nitrogen atmosphere while heating samples from 40 to 800 °C at a rate of 10 °C/min.

#### Mechanical property tests

DMA was performed on a TA Instruments Q800 in tensile mode over a temperature range of 25 to 250 °C, with a heating rate of 5 °C/min. The cross-link density was calculated according to previous literature [56] using the following formula:

$$V_{e}=\frac{E_{r}^{\prime }}{3RT_{r}}$$ (1)

where $V_{e}$ is the cross-link density, *R* is the gas constant, $E_{r}^{\prime }$ is the storage modulus at *T*_g_ + 30 °C, and *T*_r_ is the temperature (K) above *T*_g_ + 30 °C. Stress relaxation analysis was conducted using a TA Instruments Q800, employing samples with the same dimensions as those used for DMA tests. A constant strain of 1% was applied during each test, and the relaxation modulus was recorded. The stress relaxation behavior at temperatures ranging from 140 to 170 °C was investigated, and the results were fitted using a single-phase Maxwell model as follows:

$$\frac{G\left(t\right)}{G_{0}}=e^{\frac{-t}{\tau^{*}}}$$ (2)

$$\tau^{*}=\tau_{0}e^{\frac{E_{a}}{\mathrm{RT}}}$$ (3)

*G*(*t*) is the shear modulus of the material at time *t*, *G*_0_ is the initial shear modulus, and $\tau^{*}\;$ is the relaxation time, where $\;\tau_{0}$ is a material constant, *E_a_* is the activation energy, *R* is the gas constant, and *T* is the absolute temperature.

Tensile tests were performed at room temperature using a universal testing machine (Gotech AI-7000S, 5 kN) on rectangular samples (50 × 5.0 × 1.0 mm^3^). The reported results represent the average of measurements from at least 3 samples tested at a tensile rate of 10 mm/min. Toughness (*T*) is defined as the area under the engineering stress (σ) versus strain (ε) curve and was calculated as follows:

$$T=\int_{0}^{\varepsilon_{\max}}\mathrm{\sigma d\varepsilon}$$ (4)

Three-point bending tests were conducted at room temperature using a mechanical testing machine (CMT6103/Zwick/Instron 5969), with sample sizes of approximately (50 × 5.0 × 1 mm^3^). These tests were performed on 3 samples at a deflection rate of 5 mm/min until fracture. The elastic modulus (*E*) represents the ratio of stress (σ) to strain (ε) in the elastic deformation range, and was calculated according to:

$$E=\frac{\sigma }{\varepsilon }$$ (5)

To assess the processing stability, materials were processed at a 90° angle at room temperature, with angular changes recorded every 60 h.

#### Dielectric performance test

Low-frequency dielectric constant measurements were conducted using a Harbin Giant Wave TZDM-RT-1000 instrument over the frequency range of 20 Hz to 5 MHz, with sample dimensions of approximately φ10 mm × 1 mm. High-frequency dielectric constant measurements were performed using a Tektronix 4991A instrument at room temperature, using a frequency range of 2 to 3 GHz, with sample dimensions of approximately 50 mm × 50 mm × 2 mm. The dielectric strength was tested using a GJW-100E breakdown voltage instrument.

## Data Availability

The data that support the findings of this study are available within the article and the Supplementary Materials file. All other relevant source data are available from the corresponding authors upon request.
