# Supplementary material for: Processable Bio-Based Polybenzoxazine with Tunable Toughness and Dielectric Properties
Source: Research (Wash D C). 2025 Jun 24;8:0745. doi: 10.34133/research.0745 (PMC12187022; doi:10.34133/research.0745)
Supplement: Supplementary 1 — Figs. S1 to S18 Tables S1 to S10 [file research.0745.f1.docx]

**Supplementary Information**

**Processable bio-based polybenzoxazine with tunable toughness and dielectric properties**

Jiale Li^a^, Meng Liu^a^, Yuan Liu^a^, Peng Zhao^a^, Yuhan Lou^a^, Zhiqian Meng^a^, Xiaoxue Song^a,b^, Zhenle Hu^a^, Yongzhuang Liu^a^*, Haipeng Yu^a^*

*^a^Key Laboratory of Bio-based Material Science and Technology of Ministry of Education; State Key Laboratory of Utilization of Woody Oil Resource, Northeast Forestry University, Harbin 150040, China*

*^b^College of Home and Art Design, Northeast Forestry University, Harbin 150040, PR China*

*Corresponding author Email:* [*yuhaipeng20000@nefu.edu.cn*](mailto:yuhaipeng20000@nefu.edu.cn)*,* [*lyz@nefu.edu.cn*](mailto:lyz@nefu.edu.cn)

**Supplementary Figures: Figs. S1-S18**

**Supplementary Tables: Table S1-S10**

1. **Supplementary Figures**

**
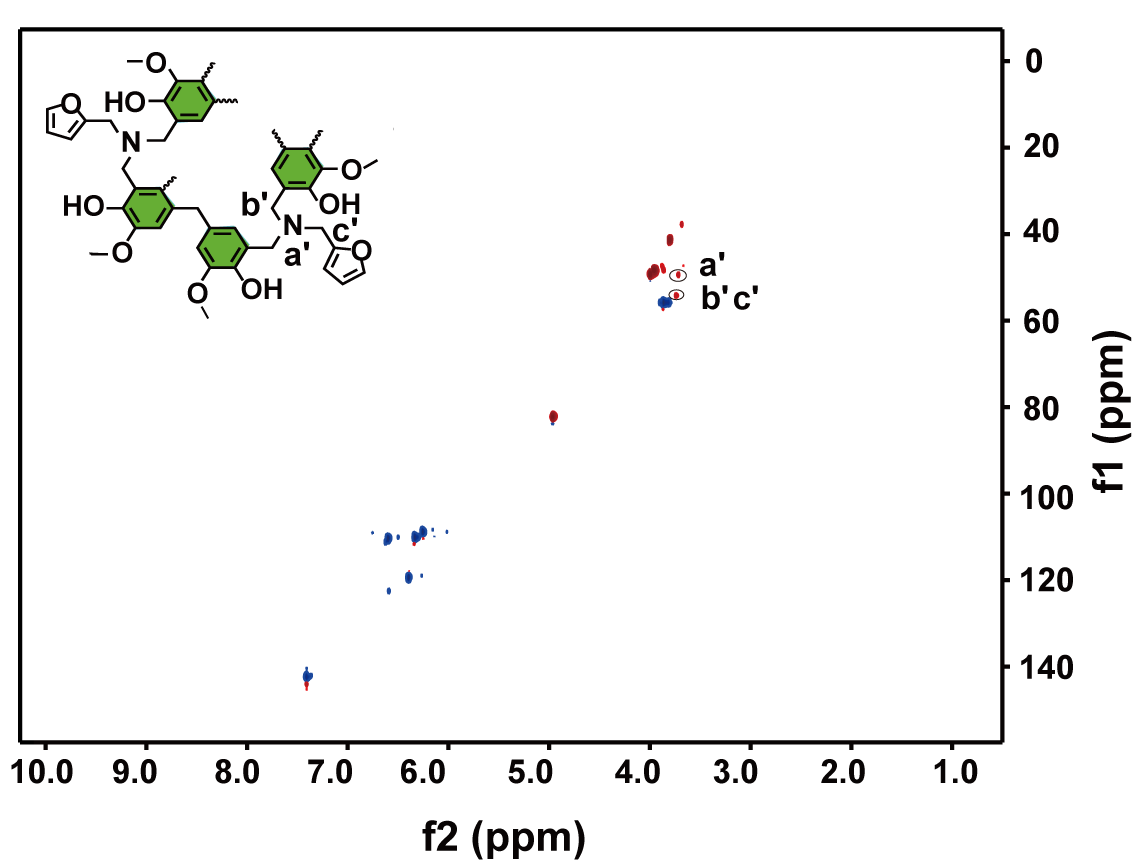
**

**Fig. S1** 2D HSQC NMR analysis of the structure and main impurities in benzoxazine dimer of BG-fa.


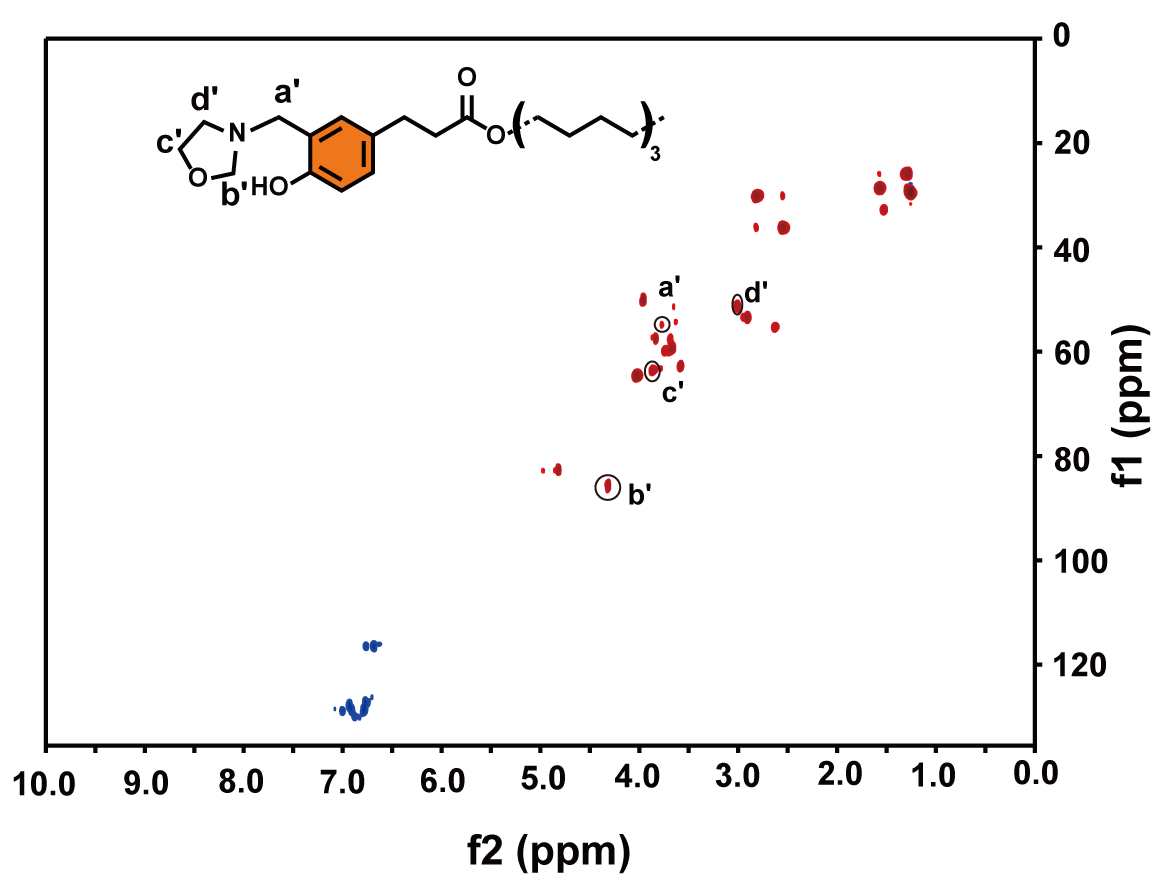


**Fig. S2** 2D HSQC NMR analysis of the structure and main impurities in benzoxazine dimer of PA-D-mea.


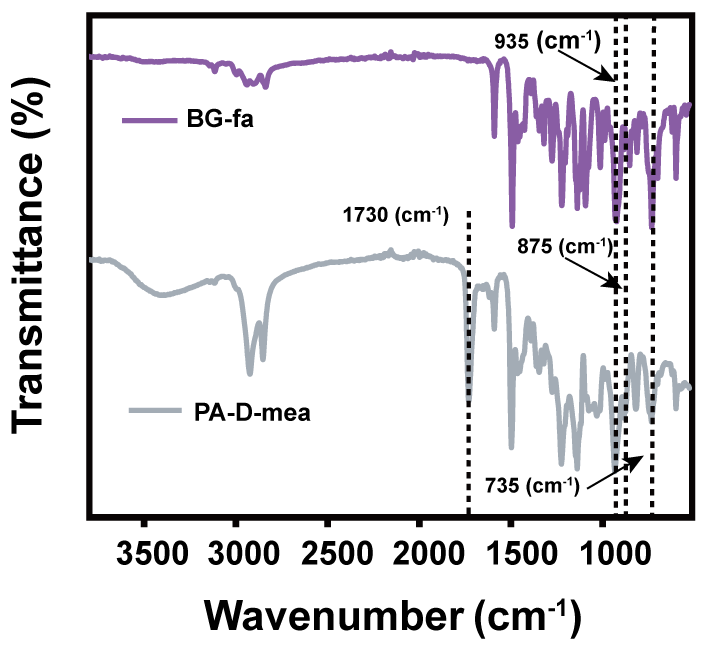


**Fig. S3** FT-IR spectra curve analysis of the dimers BG-fa and PA-D-mea.


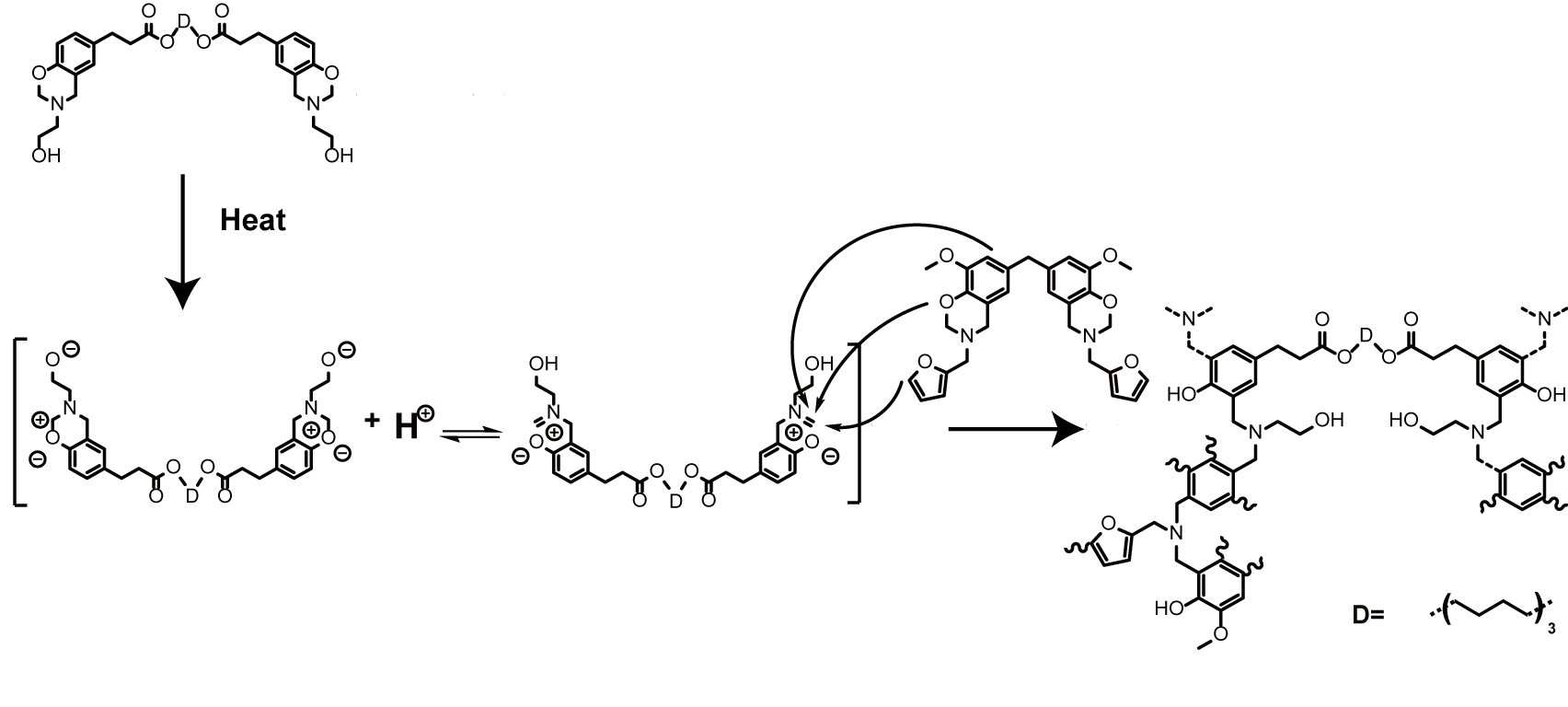


**Fig. S4** Polymerization mechanism of the copolymerization of benzoxazine dimers.


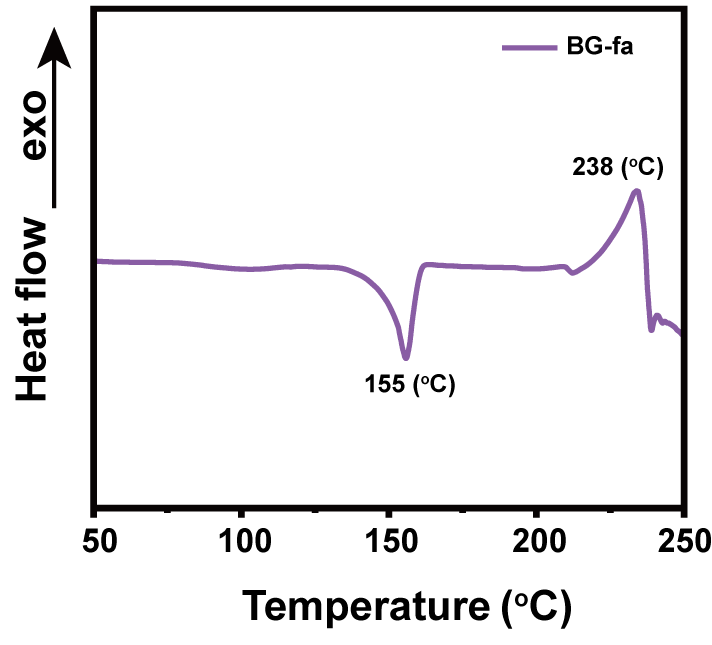


**Fig. S5** DSC curve analysis of BG-fa.


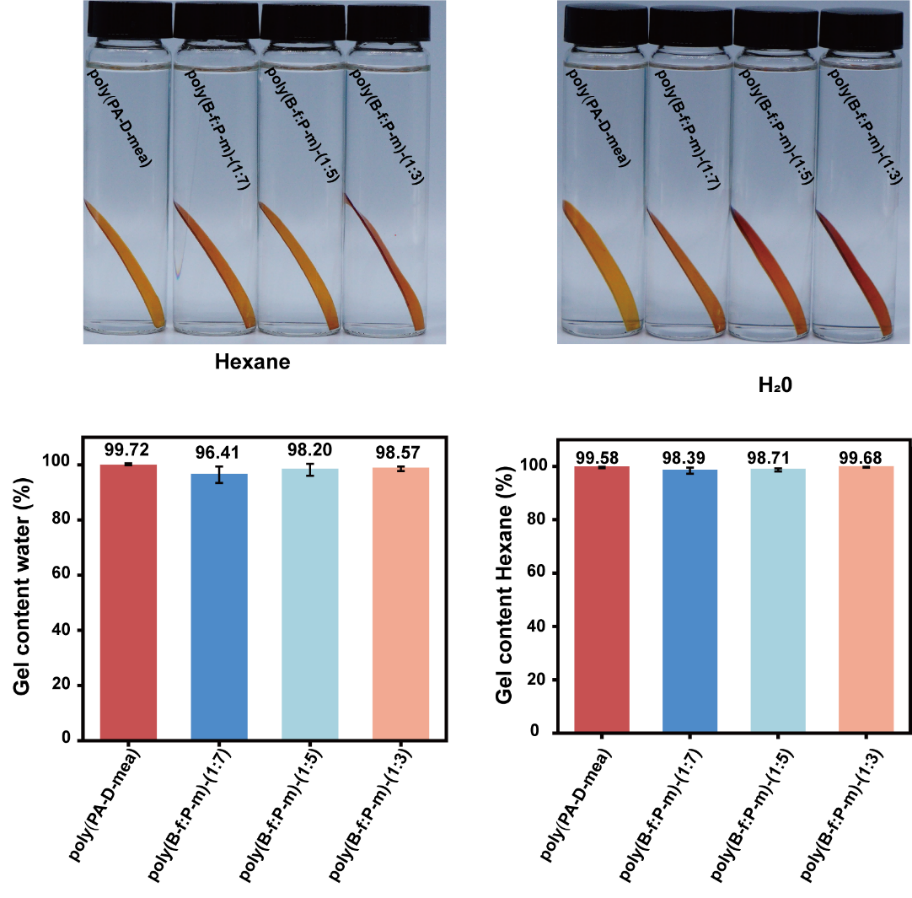


**Fig. S6** Gel content analysis of polybenzoxazine and different copolymerization ratios (1:3, 1:5, 1:7) in water and hexane.


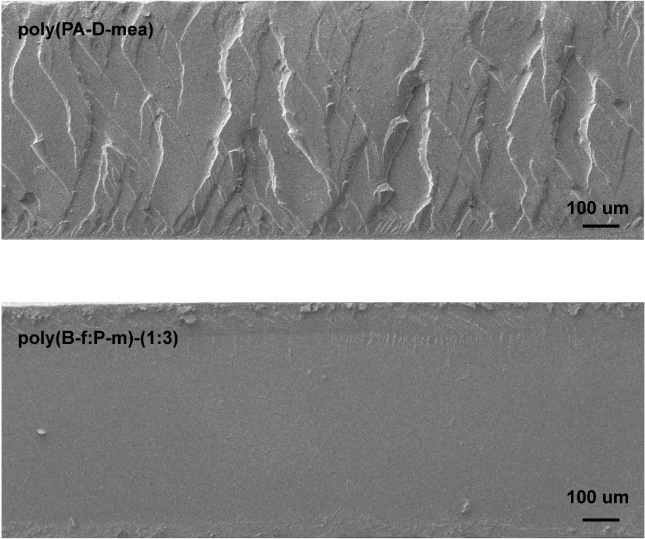


**Fig. S7** The cross-section SEM images of poly(PA-D-mea) and poly(B-f:P-m)-(1:3) at low magnification.


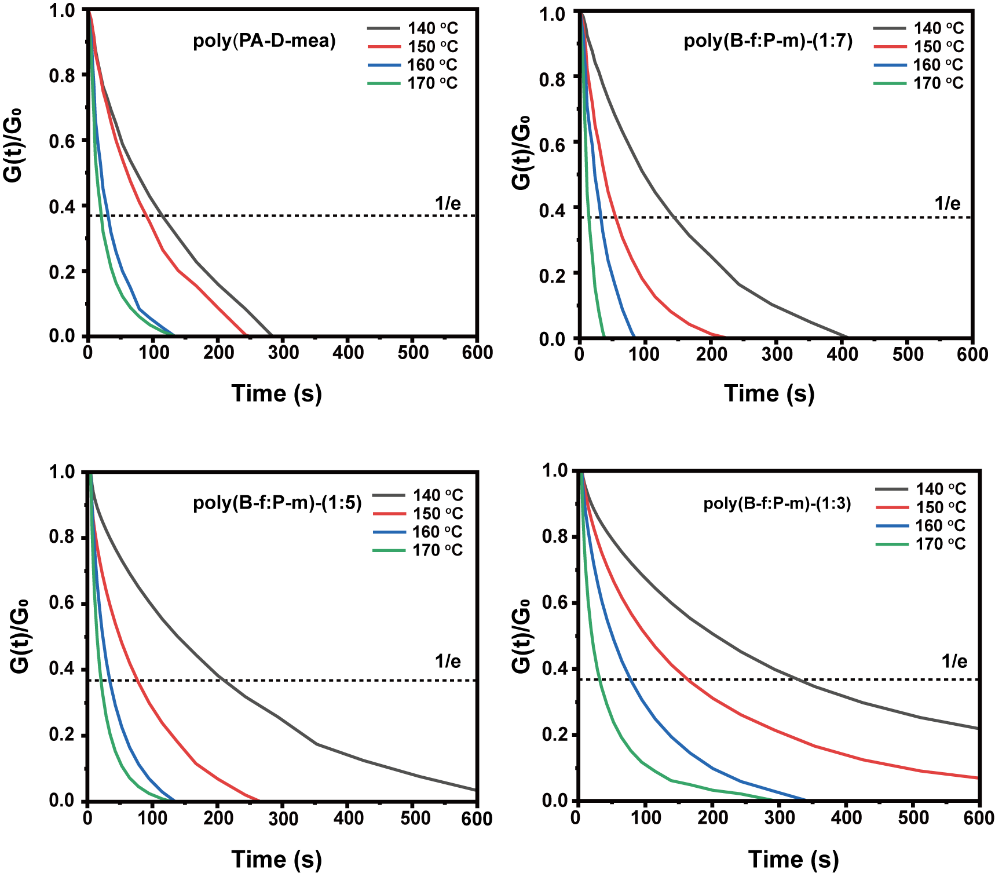


**Fig. S8** Dynamic network of stress relaxation of poly(PA-D-mea) with different copolymerization ratios (1:3, 1:5, 1:7).


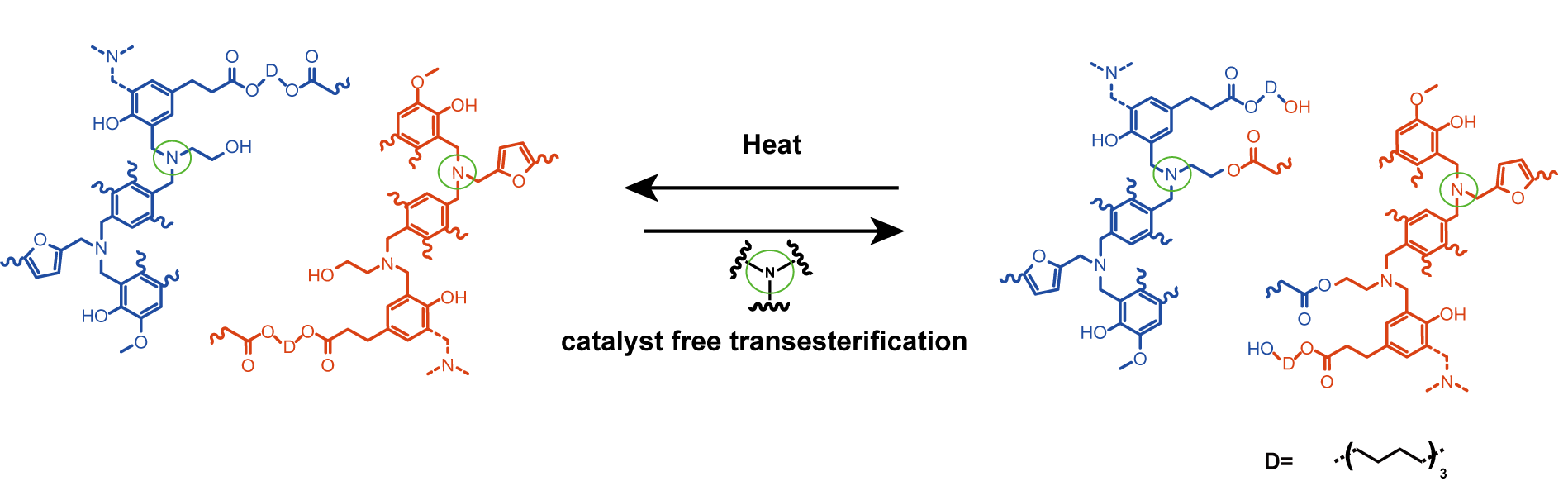


**Fig. S9** Chemical structure of internally catalyzed transesterification within polybenzoxazine.


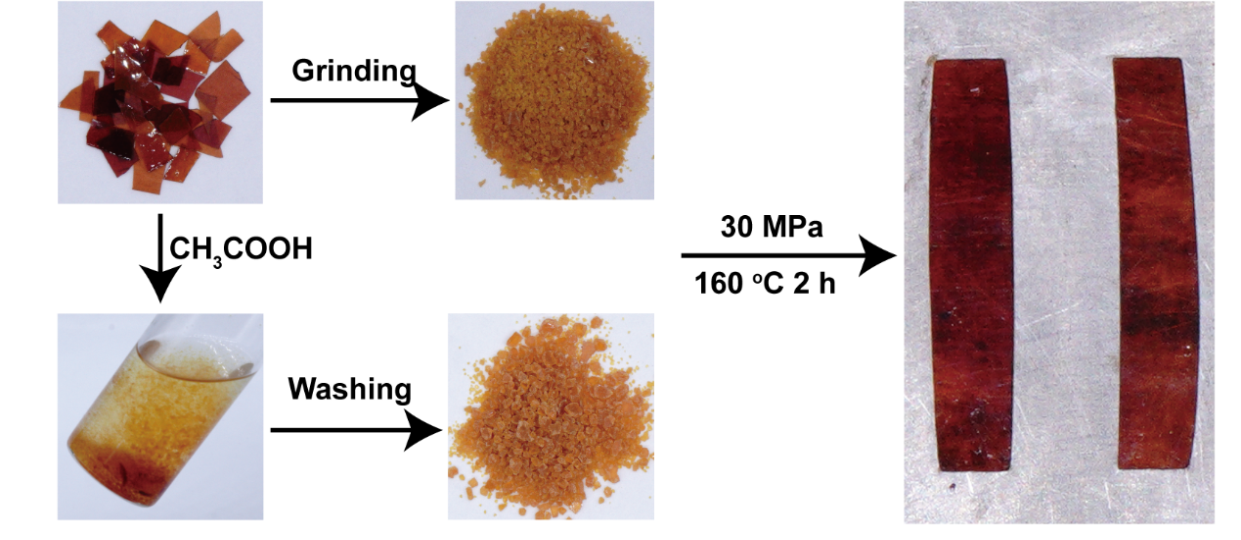


**Fig. S10** Polymer recycling by hot pressing at 160°C for 2h after physical grinding or acetic acid degradation.


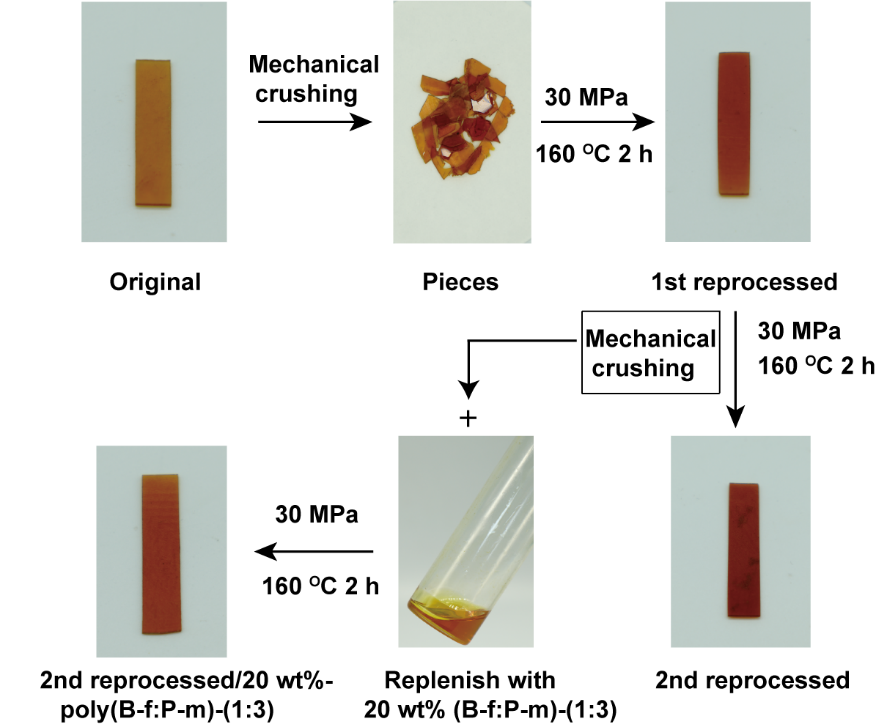


**Fig. S11** Multiple recycling cycles of poly(B-f:P-m)-(1:3) by mechanical crushing and hot pressing.


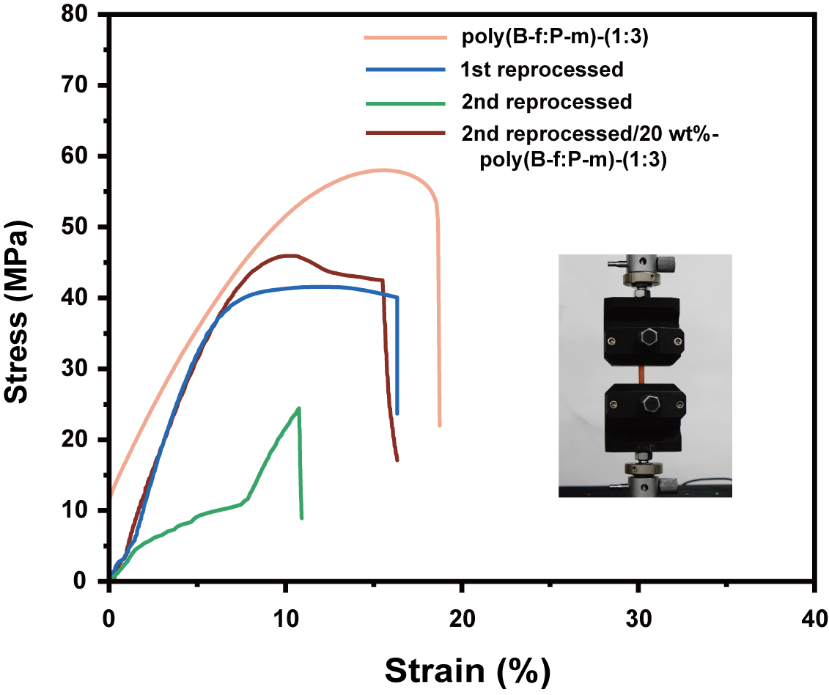


**Fig. S12** Stress-strain curves of multiple recycling cycles of poly(B-f:P-m)-(1:3) by mechanical crushing and hot pressing.


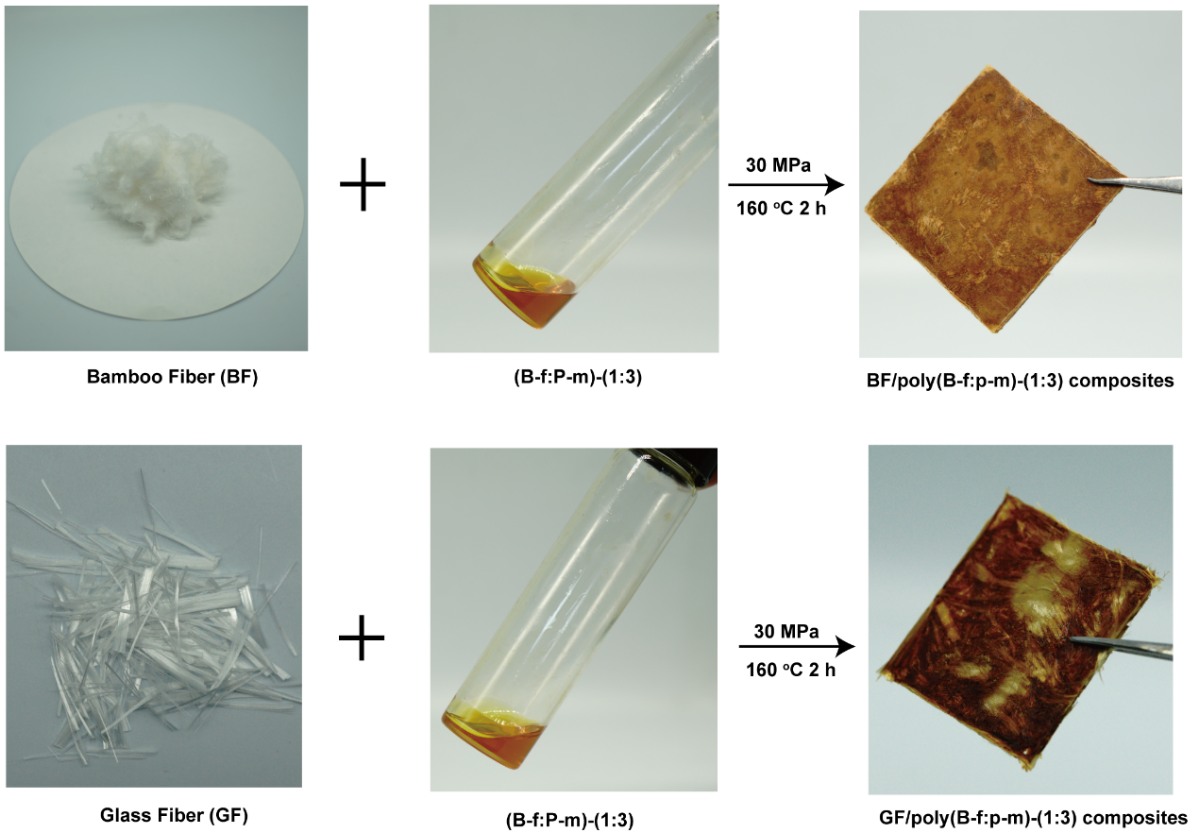


**Fig. S13** Preparation of composite material with poly(B-f:P-m)-(1:3) and bamboo fiber or glass fiber。


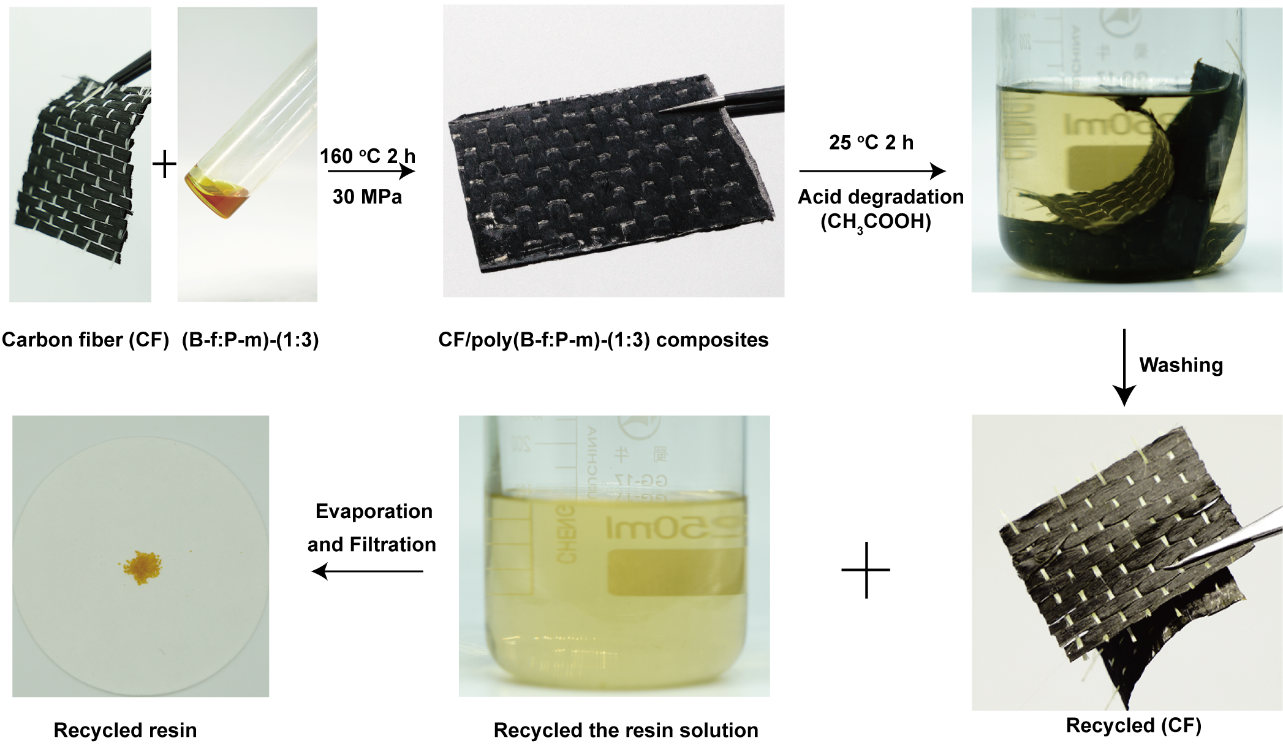


**Fig. S14** Preparation of composite material with poly(B-f:P-m)-(1:3) and carbon fiber and recycling the carbon fiber and resin by acid degradation.


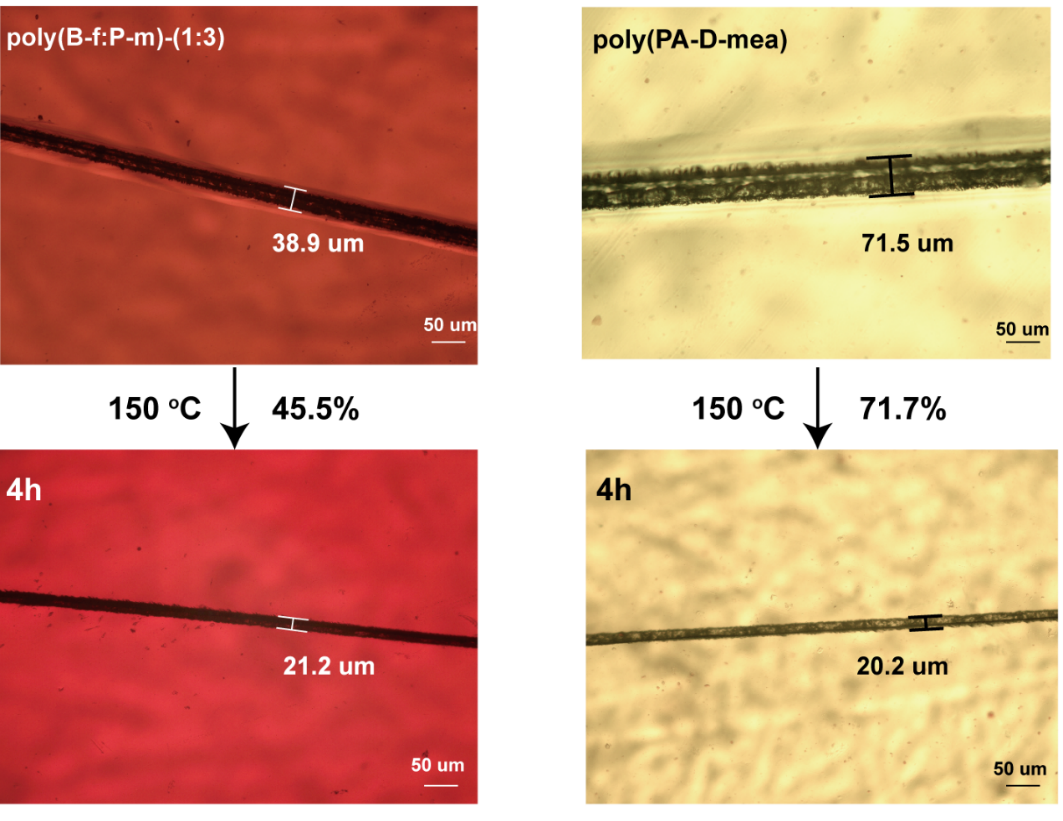


**Fig. S15** Self-healing test of poly(PA-D-mea) and poly(B-f:P-m)-(1:3) when exposed to damage at 150°C for 4h.


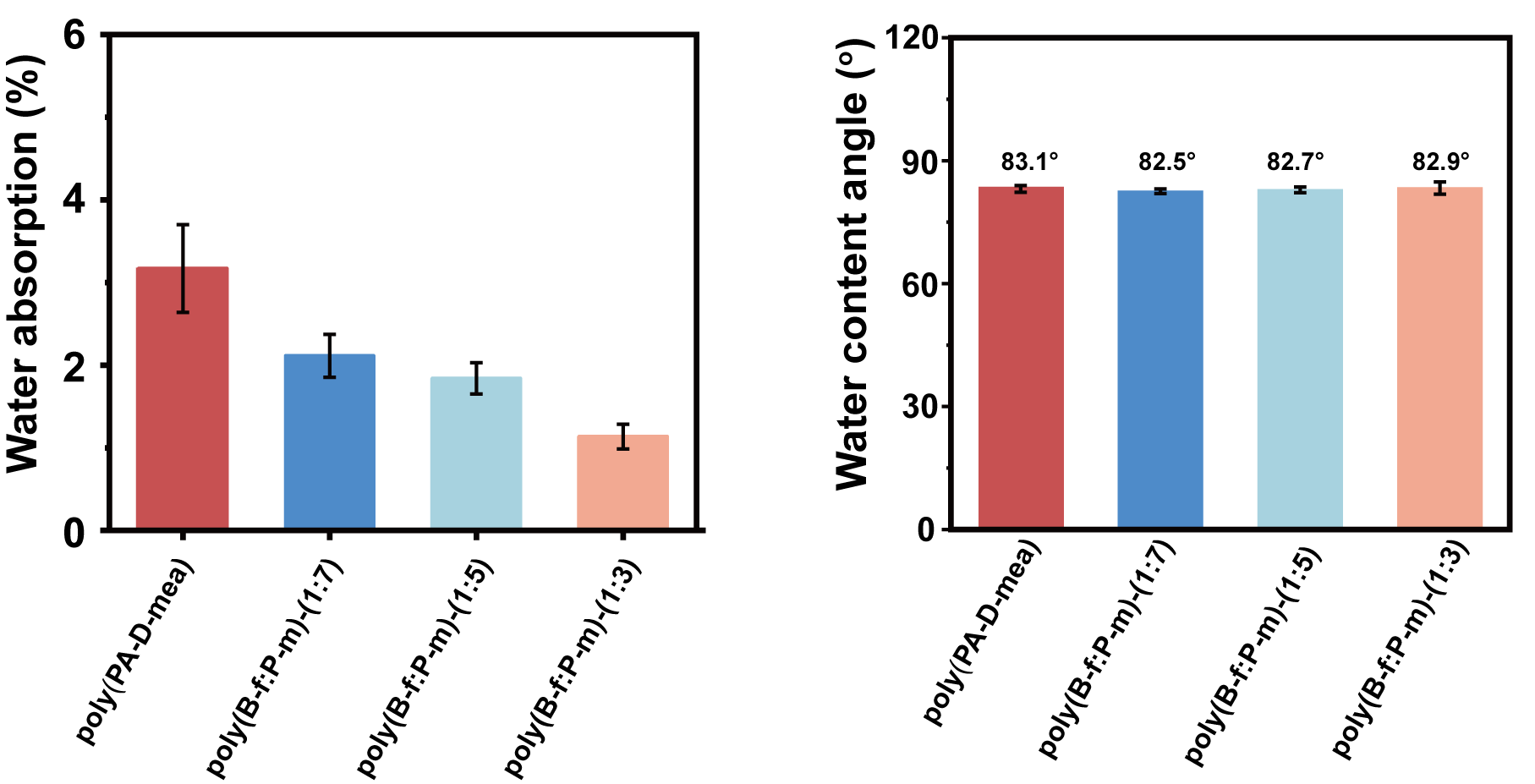


**Fig. S16** poly(PA-D-mea) with different copolymerization ratios in water absorption for one month and The liquid surface is added dropwise to maintain a contact angle for 10s.

**
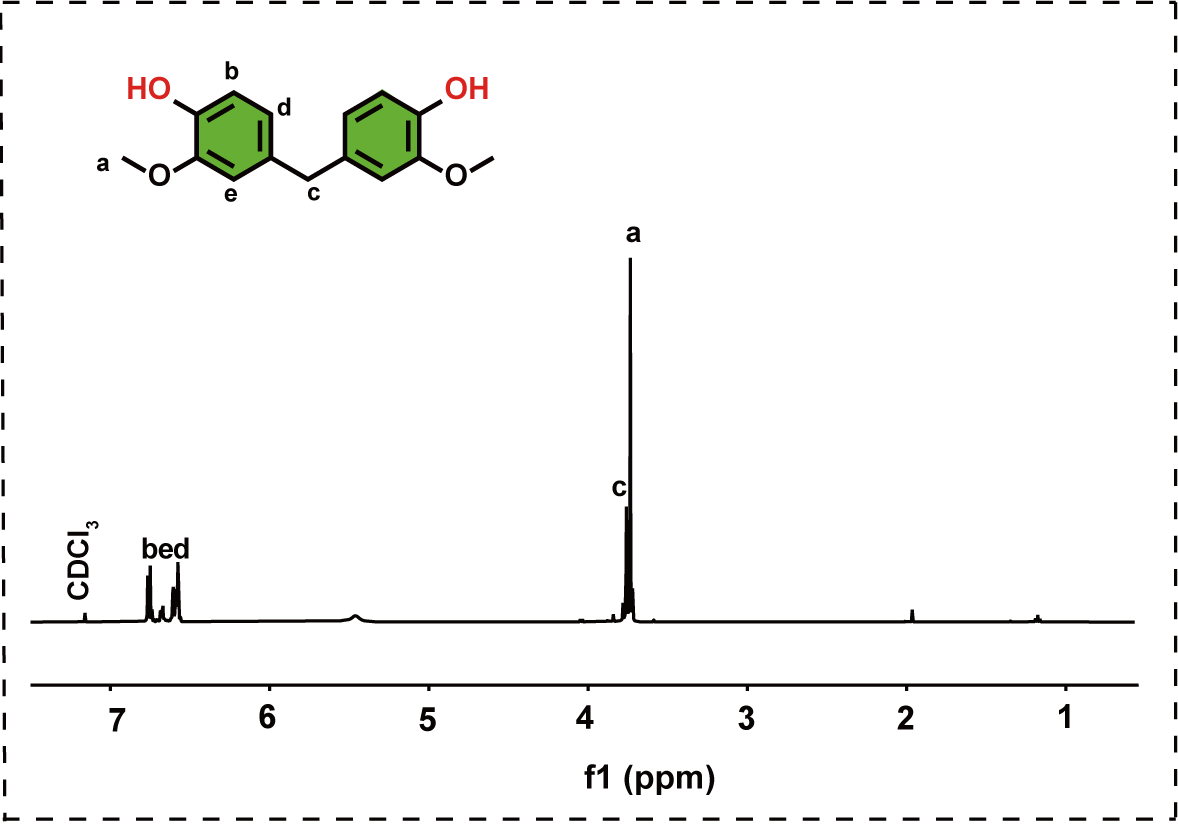
**

**Fig. S17** ^1^H NMR (deuterated CDCl_3_) spectrum of BG.


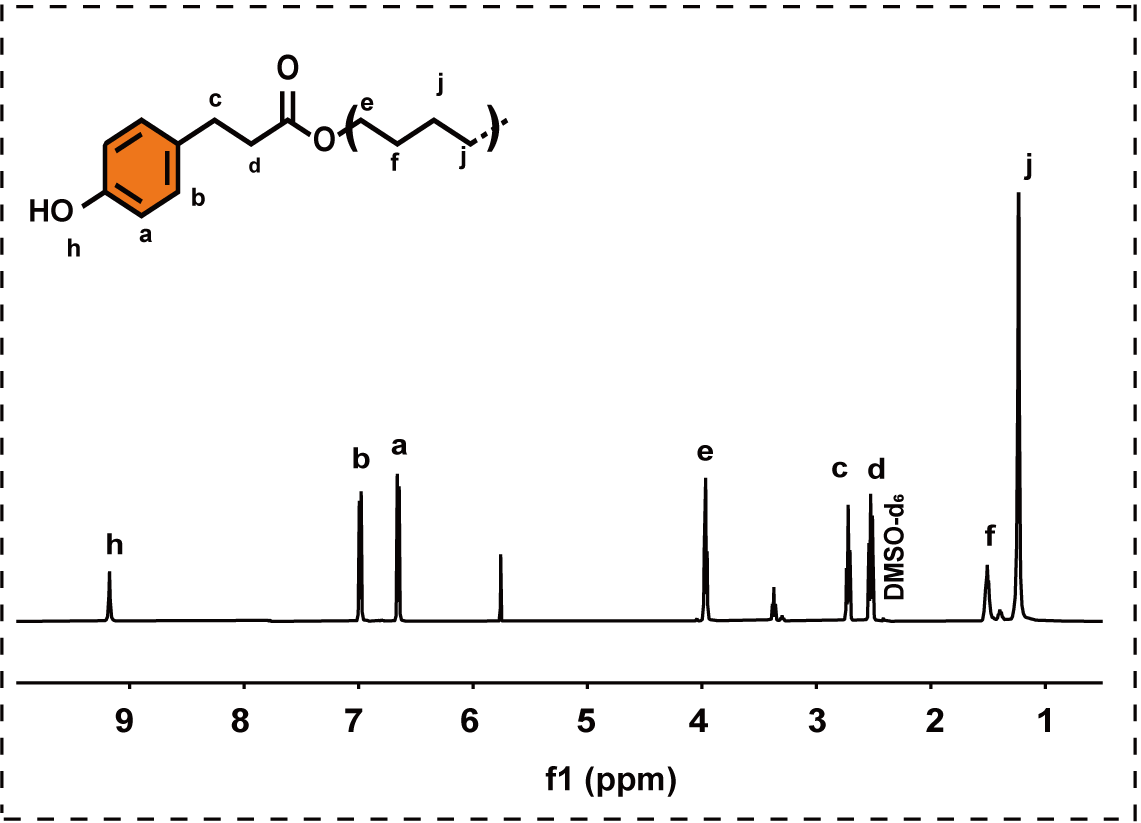


**Fig. S18** ^1^H NMR (deuterated DMSO) spectrum of PA-D.

1. **Supplementary Tables**

**Table S1** Processing temperature window and maximum ring-open loop peak temperature in DSC

| Samples | *T*_m_(℃) | *T*_0_(℃) | *T*_peak_(℃) | Processing window (℃) |
| --- | --- | --- | --- | --- |
| (PA-D-mea) | - | 170 | 222 | - |
| (B-f:P-m)-(1:3) | 108 | 157 | 219 | 49 |
| (B-f:P-m)-(1:5) | 107 | 154 | 218 | 47 |
| (B-f:P-m)-(1:7) | 106 | 159 | 217 | 53 |
| (BG-fa) | 155 | 210 | 238 | 55 |

**Table S2** Thermogravimetric properties of poly(PA-D-mea) and copolymers

| Samples | *T*_d5%_ | *T*_d10%_ | Char 800℃ |
| --- | --- | --- | --- |
| poly(B-f:P-m)-(1:3) | 270.0℃ | 298.3℃ | 29.94% |
| poly(B-f:P-m)-(1:5) | 257.4℃ | 291.8℃ | 27.56% |
| poly(B-f:P-m)-(1:7) | 254.8℃ | 287.5℃ | 24.96% |
| Poly(PA-D-mea) | 248.6℃ | 272.9℃ | 20.94% |

**Table S3** DMA properties of poly(PA-D-mea) and copolymers

| Samples | *T*_g_ (℃) | *E*'_30℃_ (MPa) | *E*' _r_ (MPa) | *V*_e_ (mol/m^3^) |
| --- | --- | --- | --- | --- |
| poly(PA-D-mea) | 78.4 | 1166.3 | 7.18 | 755.5 |
| poly(B-f:P-m)-(1:3) | 112.2 | 1775.6 | 13.69 | 1321.94 |
| poly(B-f:P-m)-(1:5) | 94.8 | 1736.3 | 11.76 | 1187.64 |
| poly(B-f:P-m)-(1:7) | 88.1 | 1525.1 | 7.51 | 770.07 |

**Table S4** Comparison of the tensile strength, elongation, *T*_g_ and processability of the polybenzoxazine in this work with the values in literature.

| **Samples** | **Tensile Strength**  **(MPa)** | **Elongation**  **(%)** | ***T*_g_**  **(ºC)** | **Processability** | **Ref** |
| --- | --- | --- | --- | --- | --- |
| poly(B-f:P-m)-(1:3) | 56.1 | 18.7 | 112.2 | **√** | This work |
| Btt-11 | 122 | 6.4 | 68 | **√** | R^1^ |
| P-AE-MV | 44.9 | 6.2 | 78.1 | **√** | R^2^ |
| p(BAEE-iBOX) | 79.5 | 11.9 | 114.8 | **√** | R^3^ |
| C-ABZ | 103.5 | 4.7 | 170 | **√** | R^4^ |
| P(DPTA-f) | 65 | 1.35 | 328 | n.s. | R^5^ |
| poly(CT-fa) | 51.6 | 1.95 | 281? | n.s. | R^6^ |
| PBAaB-0.5 | 43 | 2.04 | 178 | n.s. | R^7^ |
| poly(M-apa) | 48 | 2.15 | - | n.s. | R^8^ |
| poly(V-BZF) | 45.2 | 3.8 | 257 | n.s. | R^9^ |
| PBz-dia | 107 | 3.8 | - | n.s. | R^10^ |
| PBZ | 76.65 | 4.3 | - | n.s. | R^11^ |
| BA-a | 12.21 | 6.17 | - | n.s. | R^12^ |
| 15% BOZ/EP | 95.2 | 6.51 | 233.5 | n.s. | R^13^ |
| Poly(BAfa-70ma) | 69.7 | 7.3 | 318.6 | n.s. | R^14^ |
| poly(MBF-30E) | 94 | 11.9 | 270.6 | n.s. | R^15^ |
| poly(BT-fa) | 66 | - | - | n.s. | R^16^ |

**Btt-11:** polybenzoxazine synthesized from vanillin and furylamine and benzaldehyde (V-bfa), terephthalaldehyde (TA), and Jeffamine T403.

**P-AE-MV:** polybenzoxazine from vanillin and 1,10-diaminodecane, and blend with the epoxy resin.

**p(BAEE-iBOX):** imine-containing benzoxazine monomers after polymerization with diamines.

**C-ABZ:** polybenzoxazine vitrimer (C-ABZ) crosslinked by dynamic imine bonds.

**P(DPTA-f):** trifunctional benzoxazines by using biobased DPA, tyramine, tyrosol, and furfurylamine.

**poly(CT-fa):** polybenzoxazine synthesized from citraconic anhydride, furfurylamine and tyramine.

**PBAaB-0.5:** blends of 2,2′-Bis(4-phenyl-3,4-dihydro-2H-1,3-benzoxazinyl) isopropane (BA-a) and 4,4′-bipyridine (Bpy).

**poly(M-apa):** polybenzoxazine synthesized from magnolol with additional allyl cross-linking sites and 3-aminophenylacetylene.

**poly(V-BZF):** bio-benzoxazine made from renewable vanillin and furfuryl amine.

**PBz-dia:** polybenzoxazine containing amide groups.

**PBZ:** polybenzoxazine synthesized from cardanol, bisphenol-A, paraformaldehyde and diamines.

**BA-a:** benzoxazine resin from bisphenol A, aniline and paraformaldehyde.

**15% BOZ/EP:** copolymerization of benzoxazine and epoxy, benzoxazine containing Schiff-bases synthesized using aniline, p-hydroxybenzaldehyde, formaldehyde and pphenylenediamine.

**poly(BAfa-70ma):** polybenzoxazine from bisphenol A, melamine and furylamine.

**poly(MBF-30E):** copolymerization of melamine based-benzoxazine and epoxy resin, the benzoxazine is synthesized from bisphenol A, paraformaldehyde and melamine.

**poly(BT-fa):** polybenzoxazines synthesized from bis-thymol (BT) and furfurylamine (fa).

n.s.: now shown

**Table S5** Gel content of polymeric monomers and copolymers

| Samples | Gel Content (water)% | Gel Content (hexane) % |
| --- | --- | --- |
| poly(B-f:P-m)-(1:3) | 99.15% | 99.56% |
| poly(B-f:P-m)-(1:5) | 99.72% | 98.28% |
| poly(B-f:P-m)-(1:7) | 98.51% | 97.57% |
| Poly(PA-D-mea） | 99.64% | 99.77% |

**Table S6** Scattering factor (*q*) and spacing of periodic structures (*d*) in small-angle scattering of X-rays

|  | *q* (nm^-1^) | *d* (nm) |
| --- | --- | --- |
| poly(PA-D-mea) | 0.264 | 23.78 |
| poly(B-f:P-m)-(1:3) | 0.246 | 25.52 |

**Table S7** Mechanical properties of cured benzoxazine thermosets.

| Samples | Tensile Strength（MPa） | Toughness  (MJ/m^3^) | Flexural Strength  (MPa) | Flexural Modulus  (GPa) | Elongation  （%） |
| --- | --- | --- | --- | --- | --- |
| Poly(PA-D-mea) | 38.2±3.78 | 30.0±6.20 | 65.2±3.40 | 0.96±0.012 | 87.0±6.12 |
| poly(B-f:P-m)-(1:3) | 56.1±2.70 | 9.00±1.17 | 82.6±0.09 | 2.24±0.180 | 18.7±2.09 |
| poly(B-f:P-m)-(1:5) | 49.5±0.40 | 14.2±2.12 | 77.9±1.51 | 1.83±0.006 | 50.0±1.68 |
| poly(B-f:P-m)-(1:7) | 48.1±2.60 | 24.1±1.88 | 71.9±5.25 | 1.02±0.072 | 52.9±2.14 |

**Table S8** Activation energies from Arrhenius fit and relaxation time at 140 ℃ of the

polybenzoxazine networks

| Samples | Activation Energy (kJ/mol) | R^2^ | τ_140℃_  (s) |
| --- | --- | --- | --- |
| poly(PA-D-mea) | 95.030 | 0.94 | 114 |
| poly(B-f:P-m)-(1:3) | 118.225 | 0.99 | 326 |
| poly(B-f:P-m)-(1:5) | 116.470 | 0.98 | 209 |
| poly(B-f:P-m)-(1:7) | 115.480 | 0.98 | 143 |

**Table S9** Mechanical performance of polybenzoxazine after successive recycling cycles

| Sample | Tensile Strength  (MPa) | Strength Retention  (%) | Elongation  (%) | Elongation Retention  (%) |
| --- | --- | --- | --- | --- |
| Original | 56.1±2.70 | - | 18.7±2.09 | - |
| 1st recycle&reprocess | 40.7±2.95 | 72.5 | 16.1±2.07 | 86.1 |
| 2nd recycle&reprocess | 23.9±1.56 | 42.6 | 10.7±2.89 | 57.2 |
| 2nd recycle plus  20wt% original replenish&reprocess | 45.1±3.21 | 80.4 | 15.2±1.81 | 81.3 |

**Table S10** Electrical properties of cured benzoxazine thermosets

| Samples | Breakdown Strength  (kv/mm) | Dielectric Constant  (Low frequency) | Dielectric Loss  (Low frequency) | Dielectric Constant  (High frequency) | Dielectric Loss  (High frequency) |
| --- | --- | --- | --- | --- | --- |
| poly(PA-D-mea) | 18.08±2.44 | 4.966 | 0.055 | 3.268 | 0.041 |
| poly(B-f:P-m)-(1:3) | 27.22±1.38 | 4.038 | 0.033 | 2.990 | 0.019 |
| poly(B-f:P-m)-(1:5) | 22.85±2.26 | 4.303 | 0.042 | - | - |
| poly(B-f:P-m)-(1:7) | 21.62±2.21 | 4.501 | 0.046 | - | - |

1. **Supplementary References**

1. Wang, Z. *et al.* Influence of oxazine ring content on recyclability, shape memory, and mechanical properties of bio-benzoxazine-imine hybrid resin. *Polymer.* **307**, 127278 (2024).

2. Zhou, X. *et al.* High strength, self-healing and hydrophobic fully bio-based polybenzoxazine reinforced pine oleoresin-based vitrimer and its application in carbon fiber reinforced polymers. *Chem. Eng. J.* **484**, 149585 (2024).

3. Hamernik, L. J., Guzman, W. & Wiggins, J. S. Solvent-free preparation of imine vitrimers: leveraging benzoxazine crosslinking for melt processability and tunable mechanical performance. *J. Mater. Chem. A* **11**, 20568–20582 (2023).

4. Zhang, S. *et al.* Weldable, Reprocessable, and Water-resistant Polybenzoxazine Vitrimer Crosslinked by Dynamic Imine Bonds. *ChemSusChem* **17**, 1–9 (2024).

5. Liu, Y., Yuan, L., Liang, G. & Gu, A. Preparation of Thermally Resistant and Mechanically Strong Biomass Benzoxazine Resins via Green Strategy. *ACS Sustain. Chem. Eng.* **12**, 1247–1254 (2024).

6. Liu, Y., Yuan, L., Liang, G. & Gu, A. Synthesis of new biobased benzoxazine through green strategy and its polybenzoxazine resin with high thermal resistance and mechanical strength. *J. Polym. Sci.* **61**, 1279–1288 (2023).

7. Luan, X., Wang, B., Yang, P. & Gu, Y. Enhancing the performances of polybenzoxazines by modulating hydrogen bonds. *J. Polym. Res.* **26**, (2019).

8. Sha, X. L. *et al.* Solvent-free Synthesis of Alkynyl-Based Biobased Benzoxazine Resins with Excellent Heat Resistance. *ACS Appl. Polym. Mater.* **5**, 3015–3022 (2023).

9. Gorar, A. A. K. *et al.* Green composites from vanillin-based benzoxazine: Modified almond shell particles, curing behavior, thermal stability, mechanical properties, and stress analysis. *J. Appl. Polym. Sci.* **140**, 1–11 (2023).

10. Zhang, Y., Zhang, X., Zhan, G., Zhuang, Q. & Liu, X. Benzoxazines containing amide bonds and the toughening effect of amide bonds on polybenzoxazine. *Polym. Bull.* **82**, 1973–1984 (2024).

11. Li, X. K. *et al.* Mechanical properties, thermal stability, and mechanism of main-chain polybenzoxazine composites fabricated based on multi-scale effects and surface functionalization of graphene oxide nanosheets. *Polym. Compos.* **44**, 6758–6772 (2023).

12. Zhang, S. *et al.* How does prepolymerization affect the curing and properties of the thermosetting resins–benzoxazine resin as an example. *Prog. Org. Coatings* **189**, 108291 (2024).

13. Liu, L., Wang, F., Zhu, Y. & Qi, H. Degradable Schiff base benzoxazine thermosets with high glass transition temperature and its high-performance epoxy alloy: Synthesis and properties. *Polym. Adv. Technol.* **34**, 405–418 (2023).

14. Ye, J., Fan, Z., Zhang, S. & Liu, X. Improved curing reactivity, thermal resistance and mechanical properties of furylamine-based benzoxazine using melamine as an amine source. *Polym. Adv. Technol.* **34**, 1253–1264 (2023).

15. Ye, J., Fan, Z., Zhang, S. & Liu, X. Optimizing the dielectric and mechanical properties of melamine based-benzoxazine resin by copolymerizing with epoxy resin. *J. Appl. Polym. Sci.* **140**, (2023).

16. Mohamed Mydeen, K., Krishnasamy, B., Arumugam, H. & Muthukaruppan, A. Eco-friendly bio-based polybenzoxazine composites derived from sustainable thymol: A versatile approach and multifaceted study for enhanced applications. *J. Polym. Sci.* **62**, 3959–3978 (2024).
